# Combination therapies for the optimisation of Bispecific T-cell Engagers in cancer treatment

**DOI:** 10.1093/immadv/ltad013

**Published:** 2023-08-10

**Authors:** Winston M Zhu, Mark R Middleton

**Affiliations:** Oxford Medical School, University of Oxford, Oxford, UK; Department of Oncology, University of Oxford, Oxford, UK

**Keywords:** T-cell, bispecific, immunotherapy, combination

## Abstract

Bispecific T-cell engagers (BiTEs) redirect endogenous T-cell populations to cells expressing tumour-associated antigens to induce tumour cell killing. This inherently relies upon a cytotoxic T-cell population that is able to be recruited. In many cancers, immune checkpoints and other immunosuppressive factors in the tumour microenvironment lead to a population of anergic T-cells which cannot be redirected to tumour killing and thus impede the efficacy of BiTE therapy. Furthermore, there is evidence that BiTE therapy itself can increase immune checkpoint expression, and this is thought to be a major escape mechanism for the BiTE therapy blinatumomab. To overcome these inadequate T-cell responses, BiTEs may be combined with checkpoint inhibitors, chemotherapy, costimulatory molecules or oncolytic viruses. Study of these combinations is needed to expand the use of BiTEs in solid malignancies. This review covers the rationale, preclinical evidence and any clinical trials for these combination therapies and a few other less-studied combinations.

## Introduction

**Figure 1.**
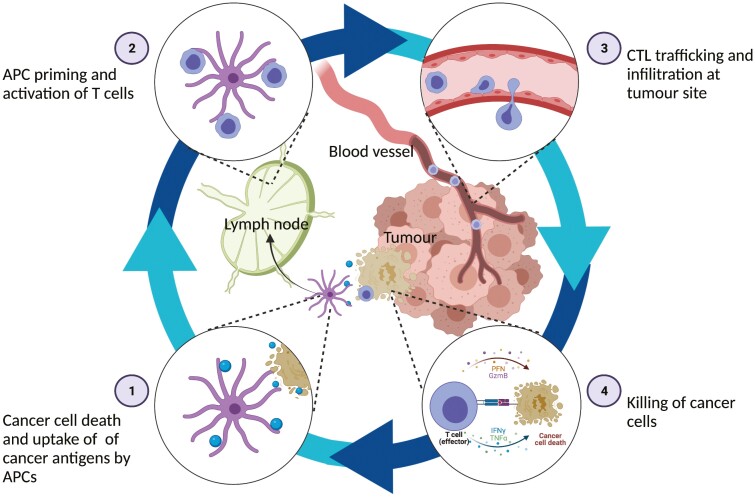
The cancer-immunity cycle (adapted from [1]). Tumour cell death leads to release of antigens which are taken up by antigen presenting cells (APCs). These travel to lymph nodes and activate lymphocytes, including cytotoxic T-lymphocytes (CTLs) which travel to the tumour site in the circulation and to the infiltrate by extravasation. These infiltrating CTLs cause tumour cell lysis which releases further tumour antigens that perpetuate the cycle. *Produced using biorender.com*.

**Figure 2.**
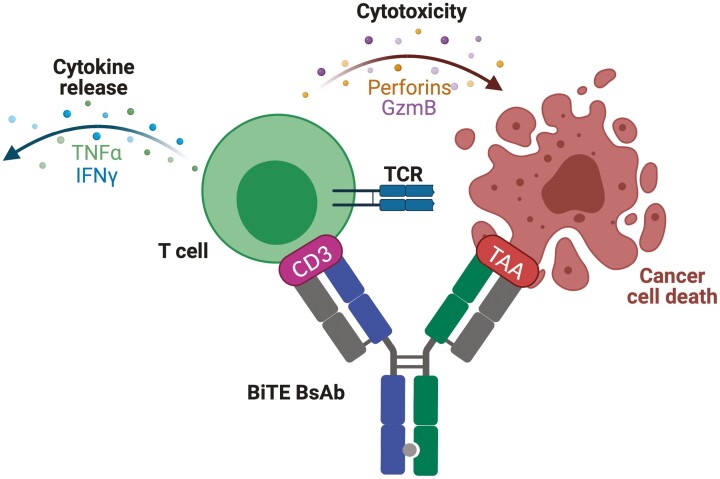
Mechanism of BiTE-mediated tumour lysis. BiTEs form an immunological synapse between T-cells and TAA presenting cells by binding CD3 (most commonly, but other T-cell-associated targets such as CD28 or 4-1BB exist, as discussed below) and a TAA of choice. Binding of both arms of the BiTE activates the T-cell and leads to degranulation of cytotoxic perforins and granzyme B across the immunological synapse, leading to tumour cell lysis [2]. As the BiTE depicted directly targets CD3 on T-cells, activation occurs in a TCR-independent fashion that results in activation of a polyclonal population of T-cells. Crucially, BiTE-mediated tumour lysis would also perpetuate the cancer-immunity (depicted in Figure 1) because activated T-cells secrete immune activating cytokines, and tumour cell lysis leads to antigen release, uptake by APCs and subsequent immune activation (Figure 1; steps 4 and 1). *Produced using biorender.com*.

Bispecific T-cell engagers (BiTEs) redirect T-cell cytotoxicity towards tumour cells, essentially kickstarting the cancer-immunity cycle described by Chen and Mellman [1] (Figure 1). BiTEs simultaneously bind a tumour-associated antigen (TAA) on one arm, and a T-cell-associated molecule (most commonly CD3) on the other (CD3xTAA). This forms an immunological synapse inducing tumour cell lysis and cytokine release (Figure 2). Because cytotoxicity is dependent on engagement of both arms of the BiTE; cytotoxicity is directed specifically towards TAA-expressing cells [2]. TAAs are also expressed on healthy tissues and so BiTEs by definition will cause some on-target off-tumour toxicity. Depending on the tumour cell type this may be tolerable, for example, the TAA CD19 is restricted to the B cell lineage and thus the CD3xCD19 BiTE blinatumomab induces tolerable and reversible B cell depletion [3]. However, many solid tumour TAAs are also expressed on a variety of healthy tissues, and although BiTE therapy in solid tumours could rely on tumour cell overexpression rather than specificity [4], on-target off-tumour toxicity remains a major barrier to BiTE therapy of solid tumours.

Many formats of BiTE exist. The first BiTE to be approved was blinatumomab, a bispecific antibody (BsAb) consisting of two single-chain variable fragments (scFvs) connected by a linker region [2]. An IgG-like structure is also commonly used. The IgG-like BiTE teclistamab has recently been approved for the treatment of multiple myeloma in the US and EU, and the IgG-like BiTE talquetamab demonstrated a 70% response rate in a phase 1 trial of heavily pre-treated multiple myeloma patients [5] and has thus been granted an expedited development pathway in the US. Immune mobilizing monoclonal T-cell receptors against cancer (ImmTACs) offer the advantage of targeting intracellular antigens via an engineered T-cell receptor (TCR) arm. The ImmTAC tebentafusp has shown a survival advantage over standard treatment in a phase 3 trial in metastatic uveal melanoma [6], and is now licensed in the US and Europe.

Blinatumomab is approved in the US and Europe for the treatment of B-cell acute lymphoblastic leukaemia (B-ALL) [7]. Side effects are generally manageable but significant adverse events include neurotoxicity and cytokine release syndrome (CRS) [3]. Despite the successes of this treatment, a significant proportion of patients are non-responders, and half of those that do respond will relapse [8]. Indeed, (BiTE-induced) oncolysis is just one step of the cancer-immunity cycle (Figure 1), and inadequacies in other steps are likely to impede therapy. For example, given the reliance of BiTE therapy on endogenous T-cell redirection, one mechanism of escape is inadequate T-cell activation [8]. This may be due to barriers such as T-cell exhaustion or the immunosuppressive tumour microenvironment (TME)—issues which can be targeted using therapeutics such as checkpoint inhibitors (CPIs). This provides a strong rationale for exploring combinations of therapies which might synergise and improve response rates for BiTEs by influencing other stages of the cancer-immunity cycle. Given that these barriers also impede the expansion of BiTE therapy to solid tumours, combination therapies may also take BiTEs beyond their current restriction to certain haematological malignancies [4].

This review focuses on the rationale, preclinical evidence, and clinical trials for the various combination therapies studied for BiTEs.

## Checkpoint inhibitors

Endogenous T-cells that are able to be redirected to tumour killing are a precondition for successful BiTE ­therapy. ­Anti-tumour activity leads to upregulation of immune checkpoints such as PD-1 and CTLA-4 on T-cells which leads to T-cell anergy and immunosuppression [9]. Furthermore, PD-L1 (PD-1 ligand) upregulation has been implicated as a mechanism of resistance to blinatumomab [10, 11]. One case study found that in a non-responder to blinatumomab, PD-L1 expression by blast cells increased from 2% pretreatment to 40% following treatment [11]. In a comparison between a non-responder and a responder to blinatumomab, PD-L1 expression was consistently higher in the non-responder blinatumomab treatment [10]. Such observations require corroboration in a larger series, but are consistent with pre-clinical observations with respect to blinatumomab resistance. One plausible mechanism for BiTE-induced PD-L1 upregulation is the release of IFN-gamma and other proinflammatory cytokines from activated T helper cells [12–14]. Indeed, BiTE-dependent PD-L1 upregulation is reduced by blockade of IFN-gamma signalling [15]. Thus, successful T-cell redirecting therapy inherently causes upregulation of the PD-1/PD-L1 axis. Furthermore, blinatumomab treatment *in vitro* induces upregulation of additional inhibitory checkpoints including CTLA4, TIM-3, and LAG-4 [16]. This begs the question as to whether PD-1/PD-L1 upregulation is an epiphenomenon or driver of BiTE resistance. Circumstantial evidence comparing responders to non-responders supports the latter. Analysis of paediatric bone marrow blasts from patients with ALL shows greater expression of PD-1 and CTLA4 in non-responders to blinatumomab compared to responders [16]. Further studies between responders and non-responders to blinatumomab, as well as longitudinal studies measuring immune checkpoint expression as patients develop resistance to blinatumomab, are required to fully understand the relationship between treatment outcomes with BiTEs and immune checkpoints.

Although a detailed mechanistic understanding is lacking, various preclinical studies have shown improved antitumor effects for CPI and BiTE in combination as compared to either therapy alone. Investigating a CD3xHER2 BiTE, Juntilla et al. found that PD-L1 expression inhibits antitumour activity by redirected T-cells *in vitro*, and this translated to an 82% rate of complete response to the CD3xHER2 BiTE in combination with anti-PD-L1 mAb in HER2+ mouse tumours compared to 43% of mice achieving a >80% reduction in tumour mass with CD3xHER2 BiTE monotherapy [17]. Whilst this clearly shows the antitumour potency of combining BiTEs and CPIs, safety must be a concern given the expression of HER2 on healthy tissues such as the heart and lungs [18], and a case report of fatal CRS during a study of CAR-T therapy targeted against HER2 [19]. BiTEs targeted against carcinoembryonic antigen (CD3xCEA) were also found to increase T-cell tumour killing with an increase in tumour cell PD-L1 expression, both *in vivo* with animal studies [20], and *in vitro* with co-cultured human tumour cells [12]. In this latter study, the authors reported improved T-cell function with anti-PD-1 and/or anti-PD-L1 therapy, an effect which is dependent on early treatment, suggesting that T-cell anergy is harder to overcome following prolonged exposure to the immunosuppressive PD-1/PD-L1 axis [12]. Flow cytometry analysis of tumours treated with a combination of CD3xCEA and anti-PD-L1 revealed a lower proportion of anergic T-cells (expressing PD-1, TIM-3, and LAG-3) compared to monotherapy, further evidencing that this combination is capable of improving T-cell functioning [15]. Preclinical evidence also supports the use of anti-PD-1/PD-L1 therapy for use with other BiTE formats, including BiTEs against Trop-2 [21] (commonly expressed in breast cancer), CEACAM5 [21], and GUCY2C [22] (expressed in colon cancers), CD33 [23] or FLT3 [24] for acute myeloid leukaemia (AML), gpA33 for colorectal cancer [25], an ImmTAC targeting NY-ESO-1 expressed in some non-small cell lung cancers [26].

CTLA-4 expression is upregulated following catumaxomab-analogue (CD3xEpCAM; BiLu) treatment [27]. Accordingly, anti-CTLA-4 treatment combined with BiLu moderately increased tumour killing and overall survival in mouse tumour models [27]. A more potent CD4+ memory T-cell response, attributable to signalling mediated by the Fc portion of BiLu, was recorded and protected animals against further tumour challenge [27].

The strong rationale and preclinical evidence for combining CPIs with BiTEs have translated into several clinical trials (Table 1). Preliminary data from a phase 1 study shows that blinatumomab in combination with the anti-PD-1 mAb nivolumab has a tolerable safety profile and achieved complete remission (CR) without minimal residual disease (MRD) in four of five patients with relapsed/refractory (r/r) B-ALL (NCT02879695) [28]. Following this result, dose escalation with ipilimumab (anti-CTLA4 mAb) will also be evaluated [28]. Preliminary data from a phase I/II trial of blinatumomab with pembrolizumab also shows a tolerable safety profile, and achieved CR in two of four evaluable patients (NCT03160079) [29]. Preliminary data from ongoing phase I studies suggest that combining a CD3xCEA BiTE with atezolizumab (anti-CTLA4 mAb) may be more effective than CD3xCEA BiTE monotherapy, and encouragingly, there was no additive effect of toxicity with this combination (NCT02650713) [30].

**Table 1.** Clinical trials combining BiTEs and CPIs. mAb: monoclonal antibody; B-ALL: B-cell acute lymphoblastic leukaemia; B-LLy B-lymphoblastic lymphoma; r/r relapsed or refractory; MRD: minimal residual disease; PRAME: preferentially expressed antigen of melanoma; ImmTAC: immune mobilizing monoclonal T-cell receptors against cancer.

BiTE	CPI	Phase	Study population	Identifier
**Blinatumomab**	**AMG404 (anti-PD-1 mAb)**	**Ib**	**r/r B-ALL**	**NCT04524455**
**Blinatumomab alone v Blinatumomab + Nivolumab**	**Nivolumab**	**II**	**First relapse B-ALL in paediatric and young adult patients**	**NCT04546399**
**Blinatumomab**	**Pembrolizumab**	**I/II**	**r/r B-ALL with high bone marrow lymphoblast percentage**	**NCT03160079**
**Blinatumomab**	**Pembrolizumab**	**I**	**r/r B-ALL in paediatric and young adult patients**	**NCT03605589**
**Blinatumomab**	**Pembrolizumab**	**Ib**	**r/r Diffuse large B-cell lymphoma**	**NCT03340766**
**Blinatumomab**	**Pembrolizumab**	**I/II**	**r/r B-ALL**	**NCT03512405**
**Cibisatamab (CD3xCEA; RO6958688)**	**Atezolizumab**	**I**	**Metastatic colorectal cancer**	**NCT02650713**
**MGD007 (CD3xgpA33)**	**MGA012 (anti-PD-1 mAb)**	**Ib/II**	**Metastatic colorectal cancer**	**NCT03531632**
**Acapatamab (CD3xPSMA)**	**Pembrolizumab**	**I**	**Metastatic castration-resistant prostate cancer**	**NCT03792841**
**REGN5678 (CD28xPSMA)**	**Cemiplimab (anti-PD-1 mAb)**	**I/II**	**Metastatic castration-resistant prostate cancer**	**NCT03972657**
**REGN7075 (CD28xEGFR)**	**Cemiplimab**	**I/II**	**Advanced solid tumours**	**NCT04626635**
**REGN4018** **(CD3xMUC16)**	**Cemiplimab**	**I/II**	**Recurrent ovarian cancer**	**NCT03564340**
**REGN5668** **(CD28xMUC16)**	**Cemiplimab**	**I/II**	**Recurrent ovarian cancer**	**NCT04590326**
**Tebentafusp** **(CD3xgp100 ImmTAC)**	**Durvalumab (anti PD-L1)** **Tremelimuumab (anti-CTLA-4)**	**Ib/II**	**Advanced cutaneous melanoma**	**NCT02535078**
**IMC-F106** **(CD3xPRAME ImmTAC)**	**Atezolizumab** **Pembrolizumab**	**I/II**	**Advanced PRAME-positive cancers**	**NCT04262466**

The synergy between BiTEs and CPIs has a strong rationale and preclinical evidence. Initial results from clinical trials are promising, but release of further results is needed to definitively show safety and efficacy. Some authors have suggested that CPIs and BiTEs should be considered as a combination therapy earlier in treatment development pipelines such that maximum tolerated dose studies of monotherapy and combination can be run in parallel [31].

## Chemotherapy

Chemotherapy may increase tumour immunogenicity and therefore acts synergistically with immunotherapy, most notably CPIs [32]. This synergy also extends to BiTE therapy, which often fails due to immune-cold environments [33]. Polyclonal T-cell recruitment is an inherent feature of CD3-targeted BiTEs, and immunosuppressive Tregs are included within this population [4]. Indeed Treg quantification was found to be an accurate predictor of response to blinatumomab [34]. Treg depletion (achievable using chemotherapy [34]) thus provides another possible mechanism for chemotherapy to synergise with BiTEs.

Preclinical data suggests that ERY974, a CD3xGPC3 BiTE, pairs synergistically with chemotherapy in mouse models of solid tumours [33]. The authors characterised one of their tumour models as non-inflamed based on low immune cell infiltration. As expected ERY974 monotherapy had little effect on this model, but the addition of chemotherapeutic agent capecitabine improved accumulation of both T-cells and ERY974 in the tumour [33]. The benefit of this combination is bidirectional as ERY974 upregulated thymidine phosphorylase, an enzyme key for the conversion of capecitabine into its active form 5-fluorouracil [33]. Capecitabine has previously been combined with other thymidine phosphorylase-inducing therapies to increase the effectiveness of chemotherapy [35], thus combination with ERY974 is a promising strategy.

For certain chemotherapy drugs, there is concern that immunosuppressive signals such as PD-L1 may be upregulated following treatment and thus hinder subsequent immunotherapy [36]. Indeed, Wathikthinnakon et al. found that gemcitabine causes upregulation of PD-L1 in cholangiocarcinoma cells [37]. As such, Wathikthinnakon et al. tested the combination of gemcitabine with a CD3xPD-L1 BiTE to turn an upregulated immunosuppressive escape mechanism into targets for BiTEs [37]. They found that gemcitabine improved BiTE-mediated T cell cytotoxicity and that this was dependent on levels of PD-L1 expression [37].

Clinical trials of BiTEs with chemotherapy have so far focussed on haematological malignancies in which both monotherapies are effective. A pivotal phase III randomised trial in 2017 showed that blinatumomab was superior to chemotherapy for B-ALL in adults in terms of overall survival, remission rates and duration of remission with similar rates of adverse events [38]. However, treatment with blinatumomab still only achieved a median duration of remission of 7 months [38]. Thus, various clinical trials are now combining blinatumomab with various chemotherapy regimes in hopes of achieving more durable remission (Table 2). Combination with blinatumomab may also allow use of lower-intensity chemotherapy regimens, sparing toxicity, and the requirement for allogenic stem cell transplantation [40, 41]. Blinatumomab has been shown to improve outcomes when combined with chemotherapy and inotuzomab-ozogamicin, a CD22-specific antibody-drug conjugate. This combination, sometimes termed condensed rituximab, inotuzomab-ozogamicin and blinatumomab (CRIB), has been shown to be effective as a salvage therapy when combined with the low-intensity chemotherapy regimen mini-hyper-CVD in several single-arm trials [42, 43], as well as superior to inotuzomab-ozogamicin or chemotherapy alone [42]. Given that these studies show more favourable outcomes when this combination is used in early salvage stages, a study of CRIB + mini-hyper-CVD as frontline therapy is warranted and underway (NCT01371630). Whilst single-group studies are useful at showing efficacy, randomised trials are needed for definitive evidence and so far only one comparing chemotherapy ± blinatumomab has reported preliminary results [44]. The ECOG-ACRIN E1910 (NCT02003222) trial compares chemotherapy ± blinatumomab as consolidation therapy following chemotherapy-induced MRD-negative remission [44]. Interim analysis reports that the addition of blinatumomab was superior to chemotherapy alone (median follow-up 43 months, blinatumomab arm median OS not reached compared to 71.4 months in the chemotherapy alone arm) [44]. The effectiveness of CRIB in combination with mini-hyper-CVD has so far not been widely reported in paediatric B-ALL populations, but a recent case study reporting MRD-negative response in one paediatric patient refractory to several other therapies, including CAR-T cell therapy, indicates a need for further study [45].

**Table 2.** Clinical trials combining BiTEs and chemotherapy. B-ALL: B-cell acute lymphoblastic leukaemia; B-LLy: B-lymphoblastic lymphoma, r/r: relapsed or refractory, hyper-CVAD: hyperfractionated cyclophosphamide, vincristine sulphate, doxorubicin hydrochloride, and dexamethasone; mini-hyper-CVD: cyclophosphamide, vincristine, and dexamethasone; MRD: minimal residual disease; PRAME: preferentially expressed antigen of melanoma; ImmTAC: Immune mobilizing monoclonal T-cell receptors against cancer.

BiTE	Chemotherapy	Phase	Study population	Identifier
**Blinatumomab**	**Chemotherapy induction as per GIMEMA LAL1913 (NCT02067143)**	**III**	**Newly diagnosed standard risk B-ALL/B-LLy**	**NCT03914625**
**Blinatumomab**	**Dexamethasone, filgrastim, pegfilgrastim, cyclophosphamide, methotrexate, cytarabine, and vincristine sulfate**	**II**	**r/r ALL**	**NCT03518112**
**Blinatumomab**	**Intozumab-ozogamicin (anti CD22 antibody-drug conjugate) and hyper-CVAD**	**II**	**Newly diagnosed B-ALL**	**NCT02877303**
**Blinatumomab**	**Condensed rituximab, intozumab-ozogamicin, and mini-hyper-CVD**	**II**	**Newly diagnosed B-ALL**	**NCT05645718**
**Blinatumomab**	**Intozumab-ozogamicin**	**II**	**Newly diagnosed or r/r CD22 positive B-ALL**	**NCT03739814**
**Blinatumomab**	**Condensed rituximab, intozumab-ozogamicin, and mini-hyper-CVD**	**II**	**Paediatric r/r B-ALL**	**NCT05645718**
**Blinatumomab**	**Intozumab-ozogamicin and mini-hyper-CVD**	**I/II**	**Frontline B-ALL**	**NCT01371630**
**Blinatumomab**	**+/- Cyclophosphamide, cytarabine, daunorubicin, dexamethasone, etoposide, mercaptopurine, methotrexate, pegaspargase, prednisone, rituximab, vincristine**	**III**	**Consolidation therapy for MRD-negative B-ALL**	**NCT02003222**
**Blinatumomab**	**Chemotherapy induction as per GIMEMA LAL1913 (NCT02067143)**	**II**	**B-ALL**	**NCT03367299**
**Blinatumomab**	**Reduced dose standard of care chemotherapy (no details given)**	**II**	**Older adults with B-ALL**	**NCT03480438**
**Blinatumomab**	**Blinatumomab and low-intensity chemotherapy (no details given) vs. standard of care alone (hyper-CVAD or as per GMALL protocol** [39]**)**	**III**	**Older adults with B-ALL**	**NCT04994717**
**Blinatumomab**	**Induction with dexamethasone, cyclophosphamide, vincristine, daunorubicin, vindesine, methotrexate, etoposide, and cytarabine followed by consolidation as per GMALL protocol** [39]**.**	**II**	**Frontline B-ALL**	**NCT04554485**
**IMC-F106** **(CD3xPRAME)**	**Chemotherapy (no details given)**	**I/II**	**Advanced PRAME-positive cancers**	**NCT04262466**

## Tyrosine kinase inhibitors

Tyrosine kinase inhibitors (TKIs), intensive chemotherapy and allogeneic stem cell transplant are the mainstay of Philadelphia chromosome-positive (Ph+) ALL [46]. Besides being two classes of drugs separately active against haematological malignancies, TKIs and BiTEs may also have some mechanistic synergy. For instance, there is evidence that ibrutinib reduces immunosuppressive PD-1 and CTLA-4 expression on T cells from chronic lymphocytic leukaemia (CLL) patients [47]. This translates into improved ex-vivo blinatumomab-induced T cell viability and cytotoxicity in CLL patient samples with ibrutinib treatment [48]. This combination is currently under investigation for r/r B-ALL in adults (Table 3; NCT02997761).

**Table 3.** Clinical trials combining blinatumomab and TKIs. Ph+ ALL: Philadelphia chromosome positive acute lymphoblastic leukaemia, B-ALL: B-cell acute lymphoblastic leukaemia, B-LLy: B-lymphoblastic lymphoma, r/r: relapsed or refractory.

BiTE	TKI	Phase	Study population	Identifier
**Blinatumomab**	**Dasatinib**	**II**	**Frontline adult Ph+ ALL**	**NCT02744768** **Follow up: NCT03318770**
**Blinatumomab**	**Dasatinib**	**II**	**Frontline adult Ph+ ALL**	**NCT04329325**
**Blinatumomab**	**Ponatinib**	**II**	**Adults over 55 with Ph+ ALL**	**NCT04688983**
**Blinatumomab**	**Ponatinib**	**III**	**Adult Ph+ ALL**	**NCT04722848**
**Blinatumomab**	**Ponatinib**	**II**	**Frontline and r/r adult Ph+ ALL**	**NCT03263572**
**Blinatumomab**	**Ponatinib and low-intensity chemotherapy**	**II**	**Adult Ph+ ALL**	**NCT03147612**
**Blinatumomab**	**Ponatinib or dasatinib**	**III**	**Frontline adult Ph+ ALL**	**NCT04530565**
**Blinatumomab**	**Dasatinib or chemotherapy**	**II**	**Adults 65 years of age or older with Ph+ ALL**	**NCT02143414**
**Blinatumomab**	**Ibrutinib**	**II**	**r/r B-ALL**	**NCT02997761**

Blinatumomab combined with dasatinib (a second-generation TKI) as a first-line treatment of Ph+ ALL in adults lead to disease-free survival of 88% at 18 months of median follow-up [49]. The ABL1 944C→T (Thr315Ile) mutation is a known driver of TKI resistance in haematological malignancies [50]. In this trial, two patients in which Thr315Ile had been detected were switched from dasatinib to the third-generation ponatinib which remains active against this mutation [49]. Recently Jabbour and colleagues have reported a phase II trial combining blinatumomab and ponatinib, a third-generation TKI active against ABL1 944C→T (Thr315Ile) variant disease which is a driver of disease relapse following initial remission—in a regimen which completely spares the patient from chemotherapy and allogeneic stem cell transplant [51]. Complete molecular response was achieved in 87% of patients with newly diagnosed Ph+ ALL and 79% of patients with r/r disease [51], warranting a randomised controlled trial of standard-of-care chemotherapy/TKI compared to ponatinib and blinatumomab. Ponatinib can cause cardiovascular adverse events which must be closely monitored for [52]. In this trial aspirin and a statin were used as prophylaxis [51].

## Costimulation

Besides signal 1 (the antigen—TCR/CD3 interaction), costimulation via a host of other pathways (signal 2), including CD28 and members of the tumour necrosis factor receptor family (such as 4-1BB, OX40, ICOS), is required for full T-cell activation [53]. As such, activation of these costimulatory pathways is an attractive method of increasing CTL activity redirected by BiTEs. A similar approach has improved the efficacy of second/third-generation chimeric antigen receptor (CAR) T-cells, which are engineered to incorporate costimulatory domains such as CD28 or 4-1BB [54].

Skokos et al. were able to develop two CD28xTAA BsAbs which were found to have little effect without signal 1 either *in vivo* or *in vitro,* but significantly increased T-cell proliferation and T-cell dependant cytotoxicity when combined with a CD3xTAA BsAbs providing signal 1 [55]. Significantly, these authors performed safety studies in cynomolgus monkeys and found that CD28xTAA did not induce systemic cytokine release, as opposed to a CD28 superagonist similar to the one which caused near-fatal CRS in 2006 [55]. This suggests that targeted CD28 costimulation, using CD28xTAA BsAbs, for example, should be focussed on in order to minimise CRS. A further safety experiment of the effect of combining CD28xTAA with CD3xTAA BsAbs in cynomolgus monkeys was not included and would be valuable. However, further data published in abstract form supports the combination of CD3xTAA BsAbs with CD3xPD-L1/B7-H3 BsAbs in terms of efficacy in tumour mouse models and safety in monkeys [56].

Using a pair of BsAbs presents the opportunity to target the same antigen by two different means (CD3xTAA1 + CD28xTAA1) or separate antigens (CD3xTAA1 + CD28xTAA2). In the former strategy, TAA arms are engineered to target different epitopes of the same antigen to prevent competition [55]. The latter strategy might improve specificity of immunotherapy given work done to profile coexpressed targets on cancer cells, for example, the combination of CD33 and TIM3 is highly specific to AML cells [57]. The BsAb pair of CD3xCD19 with CD28xPD-L1 was tested *in vitro* on B-ALL cells from patient donors, ­finding that CD28xPD-L1 BsAb alone had no effect, but did potentiate the action of CD3xCD19 [58]. PD-L1 is able to be targeted as the TAA in this combination because of the need for signal 1 (provided by CD3xCD19 BsAb) for cytotoxic effect, restricting toxicity to B-ALL cells overexpressing PD-L1 [58]. As discussed above, PD-L1 is upregulated in response to blinatumomab treatment [10, 11]. In essence, by the addition of the CD28xPD-L1, Correnti and colleagues switched an immunosuppressive signal into one which can be targeted to redirect CTLs [58]. Moreover, many factors in the TME contribute to immunosuppression via a common pathway of PD-L1 upregulation [59], but if PD-L1 is used as the TAA arm of the BiTE, this normally detrimental effect now simply increases the available targets for PD-L1 targeted BiTEs to bind [60]. This is supported by evidence from Khalique et al. showing that a PD-L1-targeted BiTE performs better in ascites fluid with an immunosuppressive TME than in a standard growth medium lacking immunosuppression [60].

A combination of CD3xTAA BsAbs with 4-1BBxTAA BsAbs has also been explored. In 2012, Hornig et al. showed the *in vitro* feasibility and improvement of combining a CD3 BiTE with antibody-ligand fusion proteins that targeted CD28 and 4-1BB activation to tumour cells [61]. T-cell proliferation and activation by a CD3xPSMA BsAb were improved by adding a 4-1BB agonist, but the authors note that a more targeted method of delivering the 4-1BB costimulation would result in lower toxicity given weight loss experienced by the mice [62]. Others have opted for antibody-ligand fusion proteins with the format 4-1BBxTAA as a method for targeted 4-1BB activation [56]. Whilst monotherapy of either CD3xCEA BsAb or 4-1BBxFAP did not control tumour growth in a mouse tumour model, a combination of these led to an increase in T-cell infiltration and inhibited tumour growth [56]. This finding was repeated using a CD3xCD20 BsAb and a 4-1BBxCD19 antibody-ligand fusion protein in a mouse model of diffuse large B-cell lymphoma [56].

Trispecific Abs (CD3xCD28xTAA) offer both signal 1 and 2 in one biologic [63]. Wu et al. were able to demonstrate the efficacy of a CD3xCD28xCD38 trispecific Ab *in vivo* against myeloma cells in mice [63]. In contrast, the same trispecific was generated with a mutant, ineffective CD28 arm, which exhibited cytotoxicity equivalent to a control Ab, demonstrating the importance of the CD28 arm in this trispecific [63]. Safety studies were also done in monkeys, showing a tolerable safety profile at doses relevant to immune stimulation [63]. The advantages of these trispecifics over BsAb pairs should be explored in terms of tumour killing, specificity/toxicity, pharmacokinetics and manufacturability.

Costimulation in combination with BiTEs shows promise in preclinical trials but as yet has not been translated into many clinical trials. A combination of CD3xMUC16 and CD28xMUC16 BiTEs is being studied in a phase I/II clinical trial (NCT04590326) for recurrent ovarian cancer, but preliminary results are yet to be reported.

## Oncolytic viruses

Oncolytic viruses (OVs) induce tumour lysis (step 1; figure 1) which promotes local T-cell response and is thus likely to synergize with BiTEs [64, 65]. A promising method of delivering this combination is by engineering the OV to express BiTEs (OV-BiTE; reviewed elsewhere [64, 66, 67]). Arming OVs with a BiTE payload may further reduce off-target toxicity, as OVs can have tropisms for specific tissues [68], may be engineered to only replicate in tumour cells [69], and can also be effectively delivered by intratumoural injection [70].

*In vitro*, evidence shows that OVs armed with a CD3xEpCAM BsAb effectively recruit endogenous T-cells to tumour killing [71]. Importantly, these authors reported no antagonistic relationship between T-cell activation and virulence, which might have been expected due to T-cell clearance of virally infected cells [71]. OVs armed with CD3xCD20 or CD3xCEA BiTEs were tested *in vivo* in mouse tumour models [69]. The OV armed with CD3xCD20 was more effective than a control OV armed with an irrelevant BiTE or BiTE alone for overall survival and CTL recruitment [69]. Furthermore, the finding that CD3xCD20 armed OV-induced PD-1 upregulation warrants further study of the addition of CPIs to this treatment regime [69]. On the other hand, the OV armed with CD3xCEA slightly increased CTL recruitment and improved survival in mouse models, but this did not differ from an OV armed with an irrelevant BiTE, suggesting that the modest therapeutic effect was attributable to the viral oncolysis alone and not the BiTE payload [69]. The authors attribute this to preexisting high CTL populations in CEA tumours (~30%), which renders other factors such as immunosuppression more relevant in this model than additional T-cell redirection using BiTEs [69]. This hypothesis also warrants further investigation with CPIs. A more recent study has armed an OV with a CD3xPD-L1 BsAb, which affects efficient tumour cell killing in *ex vivo* samples of human malignant ascites [60]. This format also induced T-cell killing of immunosuppressive tumour-associated macrophages (TAMs), further disinhibiting CTL activity [60].

OV-BiTEs are backed by the strong rationale of enhancing tumour inflammation and targeted BiTE delivery and show promising preclinical results [64, 66, 67]. These studies suggest that OV-BITE therapy might further synergise with CPIs, which are already being explored in 12 clinical trials in combination with the unarmed OV, T-VEC [67]. Preclinical studies also show that OV-BiTEs can effectively redirect T cell cytotoxicity towards stromal cancer-associated fibroblasts, which inhibit OV spread throughout the tumour and can prevent TIL recruitment [72]. OV-BITE therapy may also be combined with CAR-T-cell therapy [66], although this undermines a major advantage of BiTEs over CAR-T-cells: the former’s capacity as an off-the-shelf treatment.

## Other inhibitory pathways in the TME

This paper has focussed on CPIs, costimulation and OVs, but other immunosuppressive pathways have been identified as potential targets to combine with BiTEs in preclinical studies including galectin-1 [73, 74], CD73/A_2A_ [75], and adenosine [76]. TAA downregulation is a major predictor of BiTE efficacy [77], and thus methods of maintaining TAA expression would be a logical combination therapy to explore for BiTEs. Overexpression of IDO-1 in tumour cells leads to immunosuppression, putatively attributed to tryptophan depletion leading to T-cell dysfunction [78]. Hong et al. found that IDO-1 inhibition synergised with BiTEs targeted against EpCAM in mouse models of breast cancer [79]. However, the combination of IDO-1 inhibition and immunotherapy may be dubious given that IDO-1 inhibition combined with pembrolizumab failed to show any benefit over pembrolizumab alone in a phase 3 trial for advanced melanoma despite promising pre-clinical data [80].

Vascular endothelial growth factor (VEGF) contributes to several immunosuppressive pathways, but chiefly drives tumour angiogenesis resulting in an abnormal vascular architecture that prevents infiltration by circulating CTLs (step 3; Figure 1) [81]. Combination of anti-VEGF mAb with a CD3xGUCY2C BsAb improved tumour regression in mice xenograft models compared to either monotherapy alone [22]. This finding was associated with an increase in infiltrating CTLs [22]. Anti-VEGF has been compared to anti-PD-1 in combination with a CD3xMUC16 BsAb in preclinical studies of mouse ovarian cancer. Though both combinations performed better than their monotherapies alone, the anti-VEGF BiTE combination outperformed the CPI BiTE combination in terms of *in vivo* tumour cell killing and overall survival [82]. Given the significant number of clinical trials evaluating CPIs in combination with BiTEs (Table 1), and that a number of antiangiogenesis agents are already licensed as combinations with immunotherapies [81], future studies should evaluate the safety of anti-VEGF BiTE combinations with the aim of beginning clinical trials.

## Conclusion

**Figure 3.**
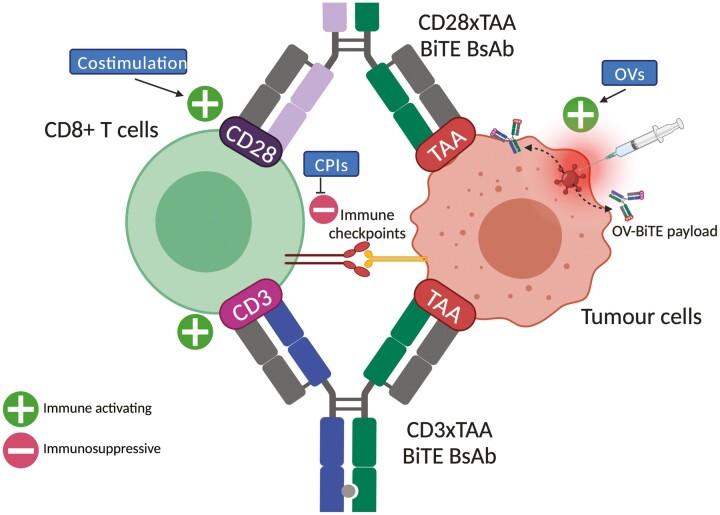
Combination therapies for BiTEs. A depiction of how BiTE combination strategies discussed. Single cell used to represent population of T cells and tumour cells for clarity. BiTE-induced tumour cell killing may be inhibited by immunosuppressive immune checkpoints, indicating that checkpoint inhibitors may synergise with BiTEs. Furthermore, pairing the traditional CD3xTAA BiTE with a CD28xTAA BiTE replicates costimulation (signal 2), leading to better T-cell activation directed towards tumour cells. Oncolytic viruses (OVs) has two distinct mechanisms. Firstly, viral oncolysis promotes inflammation (via the cancer-immunity cycle; Figure 1). Secondly, OVs can also be armed with BiTEs (OV-BiTEs), leading to selective delivery of a BiTE payload to tumour cells. OV-BiTEs may be targeted to tumour cells either by intratumoral injection (as depicted), or selective tropisms that limit replication exclusively to tumour cells. *Produced using biorender.com*.

BiTEs offer an off-the-shelf ability to redirect T-cells towards antitumour activity. As we study their capabilities and limitations in the clinic, we are constantly discovering more inhibitory pathways that impede BiTE redirection of T-cells. Furthermore, techniques including genetic analysis allow us to directly elucidate pathways that inhibit BiTE activity [77]. Identification of these pathways informs the development of new combination strategies covering potential escape mechanisms against BiTEs (Figure 3). Clearly, combining two immune-activating therapies is only useful if the safety profile of the combination is tolerable with CRS being a major dose-limiting toxicity in BiTEs [83]. In the development of future BiTEs, the choice of a CD3 targeting arm can minimise cytokine release whilst maintaining cytotoxicity [84]. Prophylactic use of an anti-IL-6 mAb, which is currently used in the management of CRS, is being trialled with CD3xPSMA BiTE for small cell lung cancer (NCT04496674) and may allow an increased maximum tolerated dose of the BiTE [85].

The hope is that combinations of BiTEs with other therapies may improve response/relapse rates for blinatumomab and expand the use of BiTEs into solid tumour treatment. So far, most preclinical studies have focused on comparisons of BiTE combination therapies against monotherapies. It is anticipated that as the field progresses and the efficacy and safety of certain combinations become more established, comparison of combinations against other combinations (as performed recently by Yeku et al. [82]) will become more of a focus, and help to inform clinical studies.

## Data Availability

No new data were generated or analysed in support of this research.

## Abbreviations

B-ALL: B-cell acute lymphoblastic leukemia
BiTE: Bispecific T-cell engager
BsAb: Bispecific antibody
CLL: Chronic lymphocytic leukemia
CPI: Checkpoint inhibitor
CRS: Cytokine release syndrome
ImmTAC: Immune mobilizing monoclonal T-cell receptors against cancer
MRD: Minimal residual disease
OV: Oncolytic virus
Ph+: Philadelphia chromosome-positive
r/r: Relapsed or refractory
ScFv: Single-chain variable fragment
TAA: Tumour-associated antigen
TCR: T-cell receptor
TKI: Tyrosine kinase inhibitors
TME: Tumour microenvironment
VEGF: Vascular endothelial growth factor
